# Platelet Count/Spleen Thickness Ratio and the Risk of Variceal Bleeding in Cirrhosis With Esophagogastric Varices

**DOI:** 10.3389/fmed.2022.870351

**Published:** 2022-07-14

**Authors:** Huimin Liu, Qun Zhang, Fangyuan Gao, Hao Yu, Yuyong Jiang, Xianbo Wang

**Affiliations:** ^1^Center of Integrative Medicine, Beijing Ditan Hospital, Capital Medical University, Beijing, China; ^2^Department of Traditional Medicine, Beijing Tsinghua Changgung Hospital, School of Clinical Medicine, Tsinghua University, Beijing, China

**Keywords:** cirrhosis, risk of esophagogastric variceal bleeding, platelet count, spleen thickness, esophagogastric varices

## Abstract

**Introduction:**

The platelet count/spleen thickness ratio (PC/ST ratio) is associated with the grade of esophagogastric varices (EGV) in cirrhotic patients, but little is known about its relationship with esophagogastric variceal bleeding (EGVB). The aim of this study was to investigate the association between the PC/ST ratio and the risk of EGVB within 1 year in cirrhotic patients.

**Methods:**

A total of 1,354 patients with cirrhosis who had EGV were enrolled in this cohort study. A logistic regression model was used to determine the association between the PC/ST ratio and the risk of EGVB within 1 year in patients with cirrhosis by adjusting the PC/ST ratio with all the important clinical variables and confounders.

**Results:**

The quartile values of the PC/ST ratio were 1.01, 1.36, and 1.98, respectively. The PC/ST ratio was an independent risk factor for variceal bleeding in cirrhotic patients with moderate or severe EGV. After adjusting for multiple variables, the relationship was still unchanged. The odds ratios of the first EGVB in these patients were 5.07-fold at non-adjustment and 3.28-fold after multivariate adjustment. The odds ratios of rebleeding in these patients from the lowest to the highest quartile were 2.34-fold at non-adjustment and 2.01-fold after multivariable adjustment. The PC/ST ratio ≤ 1.36 elevated the 1-year risk of first-time variceal bleeding or rebleeding in cirrhotic patients with moderate or severe EGV (All *P* < 0.05).

**Conclusion:**

The PC/ST ratio ≤ 1.36 is an independent risk factor for the onset of first bleeding or rebleeding in cirrhotic patients with moderate or severe EGV.

## Introduction

Esophagogastric variceal bleeding (EGVB) is a serious and fatal complication in patients with liver cirrhosis and has a high mortality rate worldwide. Esophagogastric varices (EGV) occur in nearly 50% of patients with newly diagnosed liver cirrhosis. New varices occur at the rate of 3–12% per year, and preexisting varices evolve into large varices in 8–12% of patients with cirrhosis per year (1). Combined treatment with nonselective beta-blockers (NSBB) and vasoactive drugs, endoscopic therapy, and interventional treatments are effective methods to prevent and control EGVB and are recommended for patients with acute variceal bleeding (VB) (2, 3). Although the management of portal hypertension and EGVB has significantly advanced in recent years, approximately 12% of patients still experience first bleeding each year, while rebleeding occurs in more than 20% of patients within 6 weeks (4, 5). Therefore, it is important to assess the presence of EGV and its association with the increased risk of bleeding in patients with cirrhosis.

Several studies have suggested the use of risk stratification scores in patients with EGV, which could help in assessing the risk of bleeding, prevent rebleeding, and reduce medical costs and mortality of patients (6). The hepatic venous pressure gradient (HVPG) is one of the most important indicators used to stratify patients with cirrhosis, as it can predict portal hypertension-related complications (7–9). However, HVPG is not recommended for routine use in clinical practice because of its invasive nature. Recently, various non-invasive markers have been used as predictors to evaluate the risk of first VB or rebleeding in patients with EGV, because portal hypertension depends on elevated intrahepatic vascular resistance caused due to hepatic fibrosis and cirrhotic nodules (10–13). However, the results were inconsistent due to the heterogeneity of the studies with respect to etiology, different treatments, prophylactic therapy, and cutoff values. Therefore, further studies are required to investigate and validate the association of some of the indicators with EGVB.

Previous studies have reported a correlation between the platelet count, spleen size, and EGV in patients with cirrhosis (14–19). The platelet count/spleen length ratio or platelet count/spleen diameter ratio has been validated to stratify the risk of EGV and can be used as a triage test before endoscopy to exclude adults without varices (20). Although the platelet count/spleen diameter ratio is a predictor of the existence of EGV, the relationship between it and the risk of EGVB is unknown.

We hypothesized that the platelet count/spleen size ratio may reflect the risk of the first VB occurrence in cirrhotic patients with EGV or rebleeding in patients with a history of bleeding. The present study aimed to determine whether the platelet count/spleen thickness (PC/ST) ratio can predict the risk of first-time VB and rebleeding in cirrhotic patients with EGV.

## Materials and Methods

### Study Population

This retrospective cohort study was conducted at the Beijing Ditan Hospital of Capital Medical University. The clinical data were collected from December 2008 to December 2017. A total of 1,354 patients with liver cirrhosis and EGV confirmed by the first endoscopy or endoscopic treatment were included in the analysis. All the included patients received systematic laboratory testing and ultrasound imaging, magnetic resonance imaging, or a computer tomography scan during the same period in our hospital. Written informed consent was obtained before the patient received endoscopy or endoscopic treatment. The cirrhosis was diagnosed based on as clinical symptoms, laboratory tests, and CT/MRI scan or liver biopsy (1). The exclusion criteria were as follows: (1) patients with liver carcinoma, malignancies of other organs, and other serious concurrent illnesses which causes an splenomegaly (Such as lymphoma and so on); (2) patients who received a liver transplant, splenic embolization or splenectomy, transjugular intrahepatic portosystemic shunt, esophageal and gastric fundus venous embolization and disconnection with splenectomy, or other interventions for varices; (3) patients who were lost to follow-up within 1 year or had missing data; (4) age < 20 years or > 75 years; and (5) patients without cirrhosis or having other causes of EGV. The study protocol was approved by the Ethics Committee of Beijing Ditan Hospital, Capital Medical University.

### Data Collection

Baseline clinical characteristics and laboratory data of the patients were collected at their first gastroscopy or endoscopic therapy session, including the general demographic characteristics, medical history, routine blood examination [white blood cell (WBC), red blood cell, platelet (PLT), lymphocyte, neutrophilic granulocyte, and hemoglobin (HGB)], biochemical tests [alanine aminotransferase (ALT), aspartate transferase (AST), total bilirubin (TBIL), gamma-glutamyl transpeptidase (GGT), alkaline phosphatase (ALP), albumin (ALB), and creatinine (Cr)], and coagulation function tests [prothrombin time, prothrombin activity (PTA), and International normalized ratio (INR)]. The etiology of cirrhosis was recorded. In addition, the esophageal varices were divided into slight, moderate, and severe groups according to the grading method of esophageal varices which was recommended by the World Gastroenterology Organization Global Guidelines in 2014^1^. Furthermore, abdominal ultrasonography (shape and size of the liver and spleen, and portal vein diameter) results were collected. All the ultrasound tests were performed by experienced operators. The spleen thickness was measured as follows: the splenic hilum and splenic veins were observed through the oblique intercostal section, and the diameter line from the splenic hilum to the contralateral margin of the spleen was measured as the thickness of the spleen. The PC/ST ratio, fibrosis-4 (FIB-4) score (21), model for end-stage liver disease (MELD) score (22), and aspartate aminotransferase (AST)-to-platelet ratio index (APRI) (23) were calculated using the following formulas:

PC/ST ratio = platelet count (N/mm^3^)/spleen thickness (mm)

FIB-4 = (Age × AST)/[PLT × (ALT)1/2]

MELD score = 3.78 × ln[TBiL (mg/dl)] + 11.2 × ln[INR] + 9.57 × ln[Cr (mg/dl)] + 6.43 × Etiology (Alcohol or Cholestasis: 0; Other etiologies: 1)

APRI = (AST/ULN) × 100/PLT (10^9^/L), where ULN is the upper limit of the normal AST level.

### Treatment and Endoscopic Therapy

All patients with first-time acute VB received standard treatment (2, 5), including hemostatics, vasoactive drugs (such as vasopressin, somatostatin, and their analogs), proton pump inhibitors, and endoscopic treatment at 12–24 h after hospital admission. Most patients received repeated endoscopic treatment every 3–6 months during the follow-up. Non-selective beta blockers or endoscopic variceal ligation were used for the patients with high-risk EGVB to prevent first varicose bleeding.

### Outcomes and Follow-Up

All patients were followed up for at least 1 year, and a part of patients’ follow-up ended in December 2017. The primary outcome of our study was the association between the PC/ST ratio and the risk of EGVB, including first-time VB incidence in patients with no previous VB since the detection of EGV upon their first gastroscopy, as well as variceal rebleeding incidence within 1 year in patients after suffering from first-time EGVB. We compared the abovementioned outcomes at different PC/ST ratio quartiles.

### Statistical Analyses

The baseline characteristics of the patients were compared across quartiles of the PC/ST ratio by using the Kruskal–Wallis test for non-normally distributed continuous variables, analysis of variance for normally distributed continuous variables, and the *χ^2^* test for categorical variables. Cumulative incidence of the first VB or rebleeding in each group was calculated by the Kaplan–Meier method and compared among the groups by using the log-rank test. The odds ratios for the association of the PC/ST ratio with VB were estimated using the binary logistic regression analysis models.

The PC/ST ratio was analyzed in quartiles and as a continuous variable. Important covariates for EGVB were selected on the basis of prior knowledge and statistically significant differences in the comparison among the groups. A logistic regression model was used to determine the association between the PC/ST ratio and the risk of EGVB within 1 year in patients with cirrhosis by adjusting the PC/ST ratio with all the important clinical variables and significant confounders. Analyses of the subgroups were stratified by the etiology of cirrhosis, INR, a red color sign of EGV, portal vein thrombus, and EGV grade, and the interaction among the factors was assessed by forest plots. All statistical analyses were performed using SPSS version 22.0 for Windows (SPSS, Inc., Chicago, IL, United States, RRID:BDSC_7008) and R software (version 3.6.0,^2^ RRID:SCR_002937).

## Results

### Characteristics of Patients With Esophagogastric Varices

**FIGURE 1:**
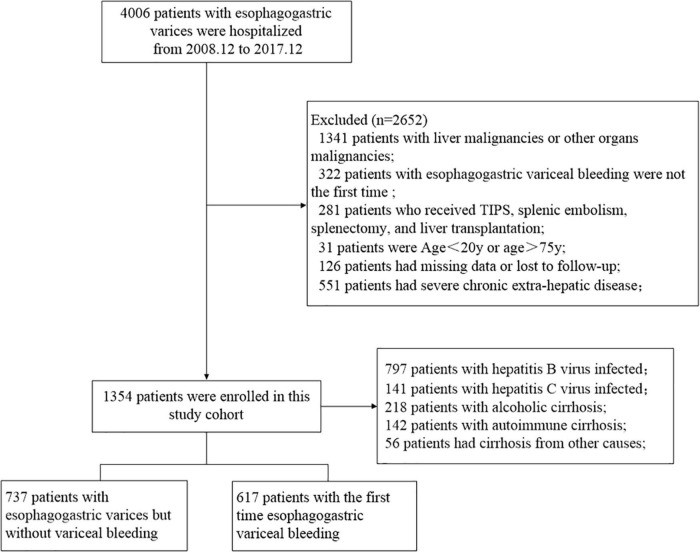
Flowchart of the enrollment of patients with liver cirrhosis and esophagogastric varices.

After excluding 1,341 patients with hepatocellular carcinoma or malignancies of other organs; 322 patients with recurrent EGVB for many years; 281 patients who received splenic embolism, splenectomy, transjugular intrahepatic portosystemic shunt (TIPS), or liver transplantation; 31 patients with age < 20 years or > 75 years; 126 patients with missing data or loss to follow-up; and 551 patients with severe chronic extra-hepatic disease, a total of 1,354 patients with liver cirrhosis and EGV were enrolled in the study. Of these patients, 938 had viral hepatitis (797 patients with chronic hepatitis B and 141 patients with chronic hepatitis C), 218 had alcoholic cirrhosis, 142 had autoimmune cirrhosis, and 56 patients had cirrhosis from other causes. Among the 1,354 patients, 737 cirrhotic patients with no previous EGVB showed EGV for the first time in gastroscopy, and 617 patients with first-time EGVB received endoscopic treatment or gastroscopy. Figure 1 shows the flowchart of the patient selection process.

The baseline characteristics of all the patients are presented in Table 1 for the overall cohort according to the PC/ST ratio quartiles. The quartile values of the PC/ST ratio were 1.01, 1.36, and 1.98, respectively. From the lowest (≤1.01) to the highest (>1.98) quartile of the PC/ST ratio, statistically significant differences were observed in the following variables: (1) patients with a lower PC/ST ratio were more likely to have cirrhosis associated with viral hepatitis and had slightly lower AST, ALT, GGT, ALP, WBC, hemoglobin, and PLT, as well as higher spleen thickness (*P* < 0.05, Table 1); (2) the incidence rates of hepatic encephalopathy, ascites, spontaneous bacterial peritonitis, and hepatorenal syndrome were higher in patients with the PC/ST ratio ≤ 1.36 (*P* < 0.05, Table 1); (3) patients with a lower PC/ST ratio (≤1.36) had a higher MELD score and hepatic fibrosis score (FIB-4 and APRI) than those with a PC/ST ratio of > 1.36, and their Child-Pugh grade was also closely related to the PC/ST ratio (*P* < 0.05, Table 1); and (4) the incidence of portal vein thrombus was also higher in patients with a lower PC/ST ratio (*P* < 0.05, Table 1).

**TABLE 1:** Characteristics of patients with esophagogastric varices according to the different platelet count to spleen thickness ratio quartiles.

	Platelet count/spleen thickness ratio quartiles (total *N* = 1,354)				*P*-value
Characteristics	Q1: ≤ 1.01 (*N* = 339)	Q2:(1.01–1.36] (*N* = 339)	Q3:(1.36–1.98] (*N* = 341)	Q4: > 1.98 (*N* = 335)	
**Demographic**					
Age	51.7 ± 10.3	52.6 ± 11.2	52.7 ± 10.8	52.3 ± 10.6	0.351
Gender (male) n (%)	188 (55.5%)	178 (52.5%)	187 (54.8%)	187 (55.8%)	0.058
**Etiology**					
HBV n (%)	222 (65.5%)	205 (60.5%)	201 (58.9%)	169 (50.4%)	<0.001
HCV n (%)	37 (10.9%)	44 (13.0%)	32 (9.4%)	28 (8.4%)	0.021
Alcoholic n (%)	43 (12.7%)	54 (15.9%)	49 (14.4%)	72 (21.5%)	<0.001
Autoimmune n (%)	19 (5.6%)	24 (7.1%)	47 (13.8%)	52 (15.5%)	<0.001
Others n (%)	18 (5.3%)	12 (3.5%)	12 (3.5%)	14 (4.2%)	0.302
**Laboratory tests**					
ALT (U/L)	29.5 (4.6–1230.5)	29.1 (1.9–1274.0)	31.5 (6.9–2493.0)	36.5 (4.7–2456.0)	<0.001
AST (U/L)	36.7 (6.1–929.2)	40.4 (9.0–1699.0)	40.5 (11.8–1595.2)	47.7 (13.5–1506.0)	<0.001
TBIL (μmol/L)	24.6 (4.5–606.5)	24.6 (0.9–489.6)	24.0 (4.5–525.0)	23.2 (3.7–408.1)	0.998
GGT (U/L)	30.0 (5.0–919.3)	40.4 (5.8–761.5)	47.8 (4.4–1065.7)	69.3 (4.7–1741.8)	<0.001
ALP (U/L)	69.4 (15.8–789.0)	80.7 (20.9–2320.0)	85.2 (17.9–678.1)	97.7 (21.7–637.7)	<0.001
Albumin (g/L)	36.20 (16.4–45.3)	32.50 (14.9–47.0)	30.96(15.8–49.3)	29.00 (12.0–38.5)	0.110
Serum creatine (μmol/L)	65.1 (7.1–522.1)	64.0 (7.4–182.4)	62.5 (11.6–268.3)	64.0 (5.28–486.9)	0.103
WBC (10^9^/L)	1.27 (0.8–50.9)	1.43 (0.8–10.30)	1.45 (0.8–10.0)	1.53 (0.6–15.0)	<0.001
Hemoglobin (g/L)	93.0 (31.1–178.0)	103.5 (32.5–170.5)	103.7 (37.2–163.0)	114.0 (35.2–176.1)	<0.001
PLT (10^9^/L)	45.1 (5.3–72.6)	59.8 (37.4–87.4)	76.0 (48.2–121.0)	113.3 (63.0–388.0)	<0.001
INR	1.3 (0.9–2.8)	1.3 (0.9–2.2)	1.3 (0.9–6.2)	1.2 (0.8–4.3)	<0.001
**Spleen thickness**	56.0 (35.0–84.0)	50.0 (29.0–71.0)	46.0 (30.0–70.0)	42.0 (23.0–77.0)	<0.001
**Portal vein thrombus**	61 (18.0%)	47 (13.9%)	46 (13.5%)	26 (7.8%)	<0.001
**Complications**					
**Hepatic encephalopathy**					
Grade I-II	29 (8.6%)	26 (7.7%)	23 (6.7%)	21 (6.3%)	<0.001
Grade III-IV	5 (1.5%)	9 (2.7%)	4 (1.2%)	2 (0.6%)	0.005
**Spontaneous bacterial peritonitis**	154 (45.4%)	134 (39.5%)	123 (36.1%)	128 (38.2%)	<0.001
**Ascites**	237 (69.9%)	219 (64.6%)	214 (62.8%)	204 (60.9%)	<0.001
**Hepatorenal syndrome**	5 (1.5%)	2 (0.6%)	1 (0.3%)	3 (0.9%)	<0.001
**Scores**					
MELD	11.0 (0.0–34.0)	10.1 (0–24.4)	10.0 (0.3–38.4)	9.9 (0–40.2)	0.001
FIB-4	8.4 (2.7–89.1)	6.6 (1.8–33.7)	5.3 (1.4–37.6)	3.6 (0.6–24.5)	<0.001
APRI	2.2 (0.4–74.2)	1.7 (0.3–57.9)	1.3 (0.4–50.5)	1.0 (0.1–26.7)	0.353
Child-pugh					
A	88 (26.0%)	94 (27.7%)	122 (35.8%)	130 (38.8%)	<0.001
B	190 (56.0%)	190 (56.0%)	175 (51.3%)	165 (49.3%)	0.032
C	61 (18.0%)	55 (16.2%)	44 (12.9%)	40 (11.9%)	<0.001

*Categorical variables are given as count (percentage), and normal and non-normal continuous variables are presented as mean ± standard deviation or median (minimum, maximum), respectively. HBV, hepatitis B virus; HCV, hepatitis C virus; ALT, alanine aminotransferase; AST, aspartate aminotransferase; TBIL, total bilirubin; ALP, alkaline phosphatase; GGT, γ-glutamyl transpeptidase; WBC, white blood cell; PLT, platelet; INR, international normalized ratio; MELD, model for end-stage liver disease; APRI, aspartate aminotransferase/platelet count ratio index.*

### A Lower Platelet Count/Spleen Thickness Ratio Corresponds to a Higher Grade of Esophagogastric Varices and Incidence of Variceal Bleeding in Patients With Cirrhosis

**FIGURE 2:**
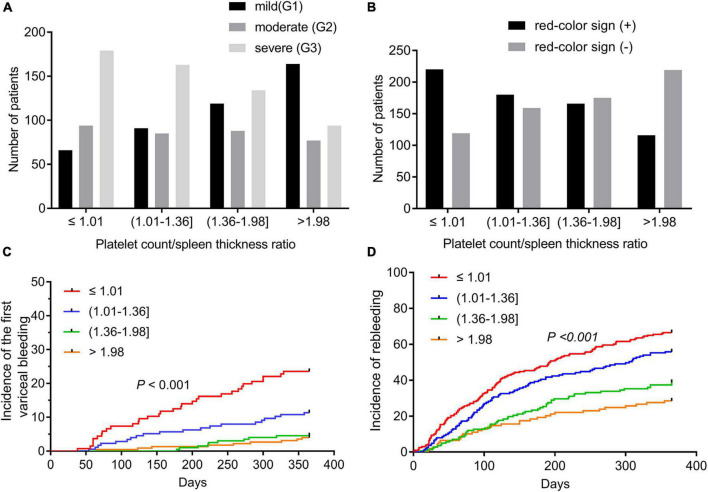
Esophagogastric varices and variceal bleeding of cirrhotic patients according to the quartiles of the platelet count to spleen thickness ratio. **(A)** Grades of esophagogastric varices in cirrhotic patients according to the quartiles of the platelet count to spleen thickness ratio. **(B)** The red color sign of esophagogastric varices according to the quartiles of the platelet count to spleen thickness ratio. **(C)** The incidence of the first variceal bleeding episode within 1 year in cirrhotic patients with esophagogastric varices according to the quartiles of the platelet count to spleen thickness ratio. **(D)** The incidence of rebleeding within 1 year in cirrhotic patients who suffered from their first EGVB according to the quartile of the platelet count to spleen thickness ratio. EGV, esophagogastric varices; EGVB, esophagogastric variceal bleeding.

Significant differences in esophageal varices grades were observed at all four PC/ST ratio quartiles (all *P* < 0.05, Supplementary Table 1 and Figure 2A). In 339 patients with a PC/ST ratio of ≤ 1.01, 19.5% had mild esophageal varices, 27.7% had moderate esophageal varices, and 52.8% had severe esophageal varices. At a PC/ST ratio > 1.98, the incidence of severe esophageal varices (28.1%) and moderate esophageal varices (23.0%) decreased. In patients with a PC/ST ratio ≤ 1.36, the red color sign of esophageal varices was > 50% (Supplementary Table 1 and Figure 2B). At 1 year of follow-up, 70 (9.5%) patients with no previous EGVB experienced their first bleeding episode, and the cumulative incidences of the first EGVB within 1 year in patients in the four PC/ST ratio quartiles were 23.5, 11.4, 4.5, and 4.0%, respectively (log-rank *P* < 0.001, Figure 2C and Supplementary Table 1). In patients with a previous first EGVB, the incidence of rebleeding within 1 year was 50.7% (313/617), and the cumulative incidences in the four PC/ST ratio quartiles were 66.5, 56.4, 38.7, and 28.4%, respectively (log-rank *P* < 0.001, Figure 2D and Supplementary Table 1).

### The Platelet Count/Spleen Thickness Ratio Is an Independent Risk Factor for Variceal Bleeding in Cirrhotic Patients With Moderate or Severe Esophagogastric Varices

**FIGURE 3:**
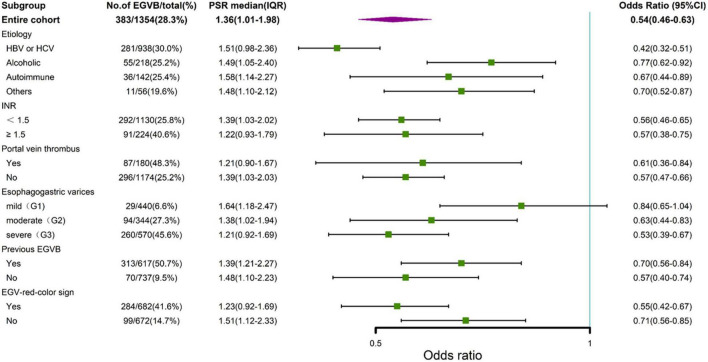
Stratified analysis by forest plots for the platelet count to spleen thickness ratio and variceal bleeding in cirrhotic patients with esophagogastric varices. EGVB, esophagogastric variceal bleeding. G1, mild grade of esophagogastric varices; G2, moderate grade of esophagogastric varices; G3, severe grade of esophagogastric varices; EGV, esophagogastric varices; 95% CI, 95% confidence intervals; PSR, platelet count to spleen thickness ratio.

The association between the PC/ST ratio and the risk of VB in cirrhotic patients with EGV might be confounded by the etiology of cirrhosis, coagulation function, EGV grade, portal vein thrombosis, the red color sign of EGV, and other factors. In stratified analyses, we found that the PC/ST ratio was an independent risk factor for variceal bleeding within 1 year in cirrhotic patients with moderate or severe EGV (mild [G1]: odds ratio [OR] = 0.84 95% confidence interval [CI] = 0.65–1.04); moderate [G2]: OR = 0.63 (95% CI = 0.44–0.83); severe [G3]: OR = 0.53 (95% CI = 0.39–0.67). Additionally, we observed that the PC/ST ratio was associated with increased risk of EGVB in patients with cirrhosis, irrespective of etiologies, INR, positive red color sign for EGV, and portal vein thrombosis (Figure 3).

### The Platelet Count/Spleen Thickness Ratio ≤ 1.36 Elevated the 1-Year Risk of First-Time Variceal Bleeding and Rebleeding in Cirrhotic Patients With Moderate or Severe Esophagogastric Varices

**FIGURE 4:**
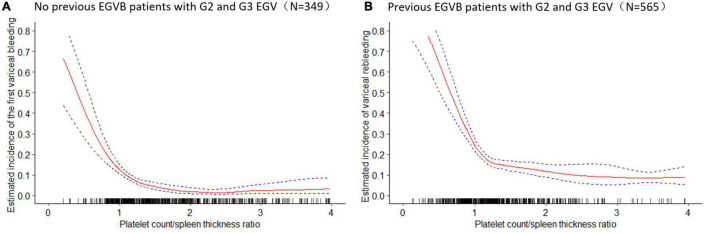
The estimated incidence of the variceal bleeding and 95% confidence intervals of variceal bleeding within 1 year in cirrhotic patients with moderate or severe esophagogastric varices. **(A)** The estimated incidence of the first variceal bleeding (%) and 95% confidence intervals of first-time variceal bleeding in cirrhotic patients with moderate and severe esophagogastric varices. **(B)** The estimated incidence of variceal rebleeding (%) and 95% confidence intervals of variceal rebleeding in cirrhotic patients with moderate and severe esophagogastric varices. EGVB, esophagogastric variceal bleeding; G2, moderate grade of esophagogastric varices; G3, severe grade of esophagogastric varices; EGV, esophagogastric varices.

To further explore the relationship between the PC/ST ratio and the moderate or severe EGV in the patients with cirrhosis, we used logistic regression analysis to determine how the PC/ST ratio influenced the risk of VB, including the PC/ST ratio alone and PC/ST ratio adjusted by multiple variables. Table 2 shows the association between the PC/ST ratio and the first bleeding episode in patients with no previous EGVB within 1 year. The unadjusted and adjusted PC/ST ratio (as a continuous) was closely associated with first-time VB in cirrhotic patients with the moderate or severe EGV, which is a significant influencing factor for these patients [Table 2, OR (95% CI), *P* value: 0.58 (0.31–0.85), *P* < 0.001 and 0.45 (0.28–0.66), *P* = 0.001]. Considering the incidence of first-time EGVB in cirrhotic patients with a PC/ST ratio (as quartiles) > 1.98 as a reference, a PC/ST ratio ≤ 1.36 (Q1 and Q2) significantly increased the risk of first-time EGVB within 1 year, regardless of whether it had been adjusted by multiple variables. The odds ratios of first-time EGVB were 5.07-fold at non-adjustment and 3.28-fold after multivariable adjustment [Table 2, OR (95% CI), *P* value: unadjusted, 5.07 (3.43–6.21), *P* < 0.001; and adjusted, 3.28 (2.40–4.17), *P* < 0.001]. An “L shaped” relationship was observed between the risk of first-time VB within 1 year and the adjusted PC/ST ratio in cirrhotic patients with moderate or severe EGV (Figure 4A).

**TABLE 2:** Adjusted effects of the platelet count/spleen thickness ratio on first-time variceal bleeding within one year in cirrhotic patients with moderate or severe esophagogastric varices.

Platelet count/Spleen thickness ratio	Odds ratios of first-time variceal bleeding (*N* = 349) (95% CI), *P*-value	
	Unadjusted	Adjusted*
Continuous	0.58 (0.31–0.85) <0.001	0.45 (0.28–0.66) 0.001
Quartiles		
Q4: > 1.98	1.00 (reference)	1.00 (reference)
Q3: (1.36–1.98]	1.08 (0.95–3.10) 0.783	1.03 (0.43–1.62) 0.869
Q2: (1.01–1.36]	3.03 (1.32–4.67) 0.007	2.28 (1.18–3.37) 0.010
Q1: ≤ 1.01	5.07 (3.43–6.21) <0.001	3.28 (2.40–4.17) <0.001

*Adjusted for age, gender, and etiology of cirrhosis, aspartate aminotransferase, alanine aminotransferase, hemoglobin, platelet, international normalized ratio, model for end-stage liver disease, red color sign of EGV, portal vein thrombosis, and esophagogastric varices grade. 95% CI, confidence interval.

Table 3 shows the relationship between the PC/ST ratio and rebleeding within 1 year after first-time EGVB in cirrhotic patients with moderate or severe EGV. The results showed that the PC/ST ratio (as a continuous variable) is an independent factor for increasing the risk of rebleeding in these patients, and this effect remained unchanged after the multivariable adjustment [Table 3; OR (95% CI), *P* value: unadjusted, 0.56 (0.44–0.70), *P* < 0.001; adjusted, 0.53 (0.42–0.67), *P* < 0.001]. Considering the incidence of rebleeding in these patients with the PC/ST ratio (as quartiles) >1.98 as a reference, the PC/ST ratio ≤ 1.36 (Q1 and Q2) significantly increased the 1-year risk of rebleeding in cirrhotic patients with moderate or severe EGV. After adjusting for multiple variables, the relationship between them still existed. The odds ratios of rebleeding from the lowest to the highest quartile were 2.34-fold without adjustment and 2.01-fold after multivariable adjustment [Table 3, OR (95% CI), *P* value: unadjusted, 2.34 (1.28–3.61), *P* < 0.001; adjusted, 2.01 (1.22–2.81), *P* = 0.006]. Figure 4B shows the relationship between the 1-year risk of rebleeding and the PC/ST ratio (adjusted).

**TABLE 3:** Adjusted effects of the platelet count/spleen thickness ratio on rebleeding within one year in cirrhotic patients with moderate or severe.

Platelet count/Spleen thickness ratio	Odds ratios of variceal rebleeding (*N* = 565) (95% CI), *P*-value	
	Unadjusted	Adjusted*
Continuous	0.56 (0.44–0.70) <0.001	0.53 (0.42–0.67) <0.001
Quartiles		
Q4: > 1.98	1.00 (reference)	1.00 (reference)
Q3: (1.36–1.98]	1.36 (0.72–1.99) 0.503	1.08 (0.63–1.53) 0.768
Q2: (1.01–1.36]	1.99 (1.25–2.74) 0.032	1.46 (1.15–1.78) 0.039
Q1: ≤ 1.01	2.34 (1.28–3.61) < 0.001	2.01 (1.22–2.81) 0.006

*Adjusted for age, gender, and etiology of cirrhosis, aspartate aminotransferase, alanine aminotransferase, hemoglobin, platelet, international normalized ratio, model for end-stage liver disease, red color sign of EGV, portal vein thrombosis, and esophagogastric varices grade. 95% CI, confidence interval.

## Discussion

Portal hypertension is the main complication experienced by patients with liver cirrhosis, and bleeding from EGV caused due to portal hypertension is a serious threat to the life of patients with cirrhosis (24). There is great clinical interest in discovering ideal and easily obtained non-invasive markers to assess the risk of EGVB and reduce the number of endoscopic therapy sessions needed for the screening and management of EGV in cirrhotic patients. Such non-invasive tools can be used to differentiate low- and high-risk EGVB patients. In the present study, we investigated the association between the PC/ST ratio and the incidence of VB in patients with cirrhosis and EGV. The analyses showed that patients with a lower PC/ST ratio (≤1.36) had a significantly increased risk of EGVB within 1 year, and the PC/ST ratio was an independent risk factor that can evaluate the risk of first-time VB and rebleeding.

In patients with cirrhosis, hypersplenism due to portal hypertension is the main cause of the reduction in platelets (25). Thus, the platelet count reflects the severity of hypersplenism and portal hypertension. Platelet count is the primary parameter in the decision rules used by hospitals in Canada to predict which patients are at low risk of variceal upper gastrointestinal hemorrhage (26). In our present study, the analysis of the non-invasive predictors was based on the maximum thickness of the spleen measured in millimeters by using abdominal ultrasound, and the platelet count. These two parameters were used to calculate the PC/ST ratio. Both these parameters are easy to obtain, non-invasive, and economical because these tests are routinely performed for patients with cirrhosis. Previous studies have reported that the platelet count/spleen diameter (or length or thickness) ratio could be used to stratify the risk of EGV in patients with chronic liver disease (19, 20), which was considered the best non-invasive predictor of EGV development (27). In the present study, we also found that the PC/ST ratio was related to several important factors of VB, including EGV grade, portal vein thrombosis, and the red color sign of EGV.

Patients with a PC/ST ratio ≤ 1.01 were more likely to have severe EGV, the red color sign of EGV, and portal vein thrombosis. The results also showed higher MELD score and hepatic fibrosis score (FIB-4 or APRI) in patients with lower PC/ST ratio (≤1.36), as well as higher incidences of hepatic encephalopathy, spontaneous bacterial peritonitis, ascites, and hepatorenal syndrome in patients with a PC/ST ratio ≤ 1.01. Thus, the PC/ST ratio represents the severity of EGV, portal hypertension, and liver cirrhosis. More than 80% of patients with cirrhosis will develop EGV (28), and the first bleeding episode occurs with 5–10% mortality after the detection of the varices (28). Endoscopy remains the only valid method to determine the presence and size of EGV. However, access to endoscopy resources and endoscopic therapy is limited in some countries, especially in those areas with a shortage of medical facilities and a strong appeal for rationalization of limited resources. Hence, identifying the risk of first-time VB is a fundamental part of the management of patients with EGV. The first crucial preventive step is to identify the patients with bleeding risk and select them for prophylactic treatment. A previous study reported that FIB-4 was a strong and significant predictor of esophageal varices, but the diagnostic accuracy for the first-time EGVB was low (10). Another recent study revealed that the liver volume index is an independent predictor of first-time VB in cirrhotic patients on propranolol prophylaxis (29), but whether this conclusion could be applied to all patients with cirrhosis remains to be validated. The findings of the present study provide evidence of a negative relationship between the PC/ST ratio and first-time VB within 1 year in patients with the lowest PC/ST ratio at the greatest risk of VB events. The risk of first-time VB was significantly increased in patients with cirrhosis who had a PC/ST ratio ≤ 1.36. With the decrease in the PC/ST ratio, the risk of first-time bleeding incidence within 1 year increased. The PC/ST ratio could be used to help clinicians to assess the risk of first-time VB, as well as improve the management and prevention of EGV in patients.

Acute EGVB is one of the serious complications of cirrhosis that leads to a high mortality rate, with a rebleeding rate of up to 60%. Treatment with NSBB and regular repeat endoscopic therapy is recommended for patients with cirrhosis who have an increased risk of EGVB (2, 30, 31). Prevention of rebleeding in these patients is one of the major goals of therapy for cirrhosis. In the present study, we found that a PC/ST ratio of ≤ 1.36 was closely associated with rebleeding of EGV within 1 year in the patients with cirrhosis. The incidence of rebleeding in cirrhotic patients increased with a decrease in the PC/ST ratio, and this effect on rebleeding did not change after adjustment with multiple variables. These findings, if confirmed by clinical trials, suggest that the assessment of the PC/ST ratio in patients with EGV could be helpful to stratify cirrhotic patients into different risk categories, identify the high risk of VB, and improve the management of patients with previous EGVB.

Our study showed that first-time VB or rebleeding risk was significantly associated with the PC/ST ratio in patients with portal hypertension, and this association remained consistent even after further adjustment with variables such as gender, age, etiology of cirrhosis, complications of cirrhosis, MELD scores, laboratory test results, the red color sign of EGV, portal vein thrombosis, and EGV grade. However, in the subgroup analysis, the VB risk was not significantly different among patients with mild EGV. This implies that the relationship between the PC/ST ratio and the risk of EGVB was significant in patients with moderate and severe EGV. The models and stratified analysis revealed an “L shape” association between the PC/ST ratio and the risk of EGVB in cirrhotic patients with moderate or severe EGV.

The present study has some limitations. First, this was a single-center study, and potential bias may be introduced due to the lack of data from multiple centers. Second, the present study investigated the relationship between the PC/ST ratio and 1-year EGVB; however, the value of PC/ST ratio for assessing the long-term risk of EGVB in patients with cirrhosis is unknown. We will continue to explore the effect of PC/ST ratio on long-term EGVB in patients with cirrhosis in a prospective study with multiple centers.

## Conclusion

In conclusion, the PC/ST ratio ≤ 1.36 is associated with EGVB in cirrhotic patients with moderate or severe EGV and should be considered when identifying patients with cirrhosis who have a high risk of VB. The PC/ST ratio may help in developing a more effective and useful non-invasive method to assess the bleeding risk of patients with moderate and severe EGV, which may further help to improve the management of patients with portal hypertension.

## Data Availability Statement

The raw data supporting the conclusions of this article will be made available by the authors, and further inquiries can be directed to the corresponding authors.

## Ethics Statement

The studies involving human participants were reviewed and approved by the Ethics Committee of Beijing Ditan Hospital, Capital Medical University. The patients/participants provided their written informed consent before the endoscopy procedure in this study.

## Author Contributions

XW and YJ participated in designing the research plan and revised the draft. HL designed the research plan, analyzed data and wrote this manuscript. QZ contributed in collecting all clinical data of patients and was involved analysis of data. FG and HY participated in the collection of clinical data. All authors listed in the manuscript have contributed to this study and approved of the manuscript being submitted.

## Conflict of Interest

The authors declare that the research was conducted in the absence of any commercial or financial relationships that could be construed as a potential conflict of interest.

## Publisher’s Note

All claims expressed in this article are solely those of the authors and do not necessarily represent those of their affiliated organizations, or those of the publisher, the editors and the reviewers. Any product that may be evaluated in this article, or claim that may be made by its manufacturer, is not guaranteed or endorsed by the publisher.

## Footnotes

1
https://www.worldgastroenterology.org/guidelines/esophageal-varices/esophageal-varices-english

2
http://www.r-project.org

## Abbreviations

EGVB: esophagogastric variceal bleeding
EGV: esophagogastric varices
VB: variceal bleeding
HVPG: hepatic venous pressure gradient
PC/ST: platelet count/spleen thickness
TIPS: transjugular intrahepatic portosystemic shunt
MELD: model for end-stage liver disease
TBIL: total bilirubin
ALT: alanine aminotransferase
AST: aspartate aminotransferase
INR: international normalized ratio
WBC: white blood cell
HGB: hemoglobin
PLT: platelet
MELD: model for end-stage liver disease
EGV: esophagogastric varices
95% CI: 95% confidence interval
GGT: gamma-glutamyl transpeptidase
ALP: alkaline phosphatase
PTA: prothrombin activity
NSBB: nonselective beta-blockers.
