# Supplementary material for: Platelet Count/Spleen Thickness Ratio and the Risk of Variceal Bleeding in Cirrhosis With Esophagogastric Varices
Source: Front Med (Lausanne). 2022 Jul 14;9:870351. doi: 10.3389/fmed.2022.870351 (PMC9329511; doi:10.3389/fmed.2022.870351)
Supplement: Supplementary file 1 [file Table_1.DOCX]

| **Table S1. Incidence of esophageal varices grades and variceal bleeding of cirrhotic patients according to quartiles of the platelet count to spleen thickness ratio.** | | | | | | |
| --- | --- | --- | --- | --- | --- | --- |
| **Characteristics** | **Total**  **(N=1354)** | **Platelet count/spleen thickness ratio quartiles** | | | | ***P* value** |
|  |  | **Q1: ≤ 1.01**  **(N=339)** | **Q2:(1.01-1.36**]  **(N=339)** | **Q3:(1.36-1.98**]  **(N=341)** | **Q4: > 1.98**  **(N=335)** |  |
| **Oesophageal varices grades** | |  |  |  |  |  |
| mild (G1) | 440(32.5%) | 66(19.5%) | 91(26.8%) | 119(34.9%) | 164(49.0%) | ＜0.001 |
| moderate (G2) | 344(25.4%) | 94(27.7%) | 85(25.1%) | 88(25.8%) | 77(23.0%) | ＜0.001 |
| severe (G3) | 570(42.1%) | 179(52.8%) | 163(48.1%) | 134(39.3%) | 94(28.1%) | ＜0.001 |
| **Red-color sign** | 682(50.4%) | 220(64.9%) | 180(53.1%) | 166(48.7%) | 116(34.6%) | ＜0.001 |
| **Variceal bleeding** | 407(30.1%) | 159(48.1%) | 107(31.5%) | 81(23.8%) | 60(17.9%) | ＜0.001 |
| The first bleeding | 70/737(9.5%) | 32/136(23.5%) | 20/176(11.4%) | 9/199(4.5%) | 9/226(4.0%) | ＜0.001 |
| Rebleeding | 313/617(50.7%) | 135/203(66.5%) | 92/163(56.4%) | 55/142(38.7%) | 31/109(28.4%) | ＜0.001 |
| Categorical variables are given as count (percentage).  . | | | | | | |
